# Western New York Dental Association

**Published:** 1868-02

**Authors:** L. J. Walter, W. C. Barrett

**Affiliations:** Warsaw, N. Y.; Warsaw, N. Y.


### SUBJECTS FOR DISCUSSION.

### WESTERN NEW YORK DENTAL ASSOCIATION.

### SEMI-ANNUAL CALL.

The Sixth Semi-Annual Meeting of this Association will be held in the hall of the Buffalo Medical Society, Young Men's Association Buildings, in the city of Buffalo, on Tuesday, May 5th, 1868, commencing at ten o'clock, A. M., and continuing two days. It is earnestly requested that all members will be present, as business of unusual importance will claim the attention of the Association.

There will be clinics in Operative and Mechanical Dentistry. Demonstrations of new and improved methods of practice, with open and free discussion of such subjects as may be presented. All practicing Dentists, in good standing, are welcomed to membership. The Medical Profession are cordially invited to be present, and to join in the discussions.

SUBJECTS FOR DISCUSSION.

1. The Influence of the Habits and Food of the Mother upon the Growth and Health of the Teeth of the Child; Essayist, Dr. B. T. Whitney. 2. Treatment of Imperfect Dentition and Irregularities of the Teeth; Essayist, Dr. G. C. Daboll. 3. Operative Dentistry; Essayist, Dr. J. C. Gifford. 4. Mechanical Dentistry; Essayist, Dr. B. Rathbone. 5. Miscellaneous Subjects; Essayist, Dr. C. W. Stainton.

L. J. WALTER, *President.*

W. C. BARRETT, *Secretary.*

Warsaw, N. Y., March 15th, 1868.

